# Dr. Barry, on the Hot Bath

**Published:** 1800-08

**Authors:** John Milner Barry

**Affiliations:** Cork


### *To Dr.* BRADLEY.

DEAR SIR,

I Thank you for the Cow-pox matter, with which I began to inoculate on the 6th inſtant; and have the pleaſure to inform you, that moſt of the children have taken the infection. I encloſe a caſe, which, with the remarks, I hope you will think worthy of a place in your valuable Journal. I am,

DEAR SIR,
Your obliged and faithful ſervant,
JOHN MILNER BARRY, M.D.*

*Cork, June* 18, 1800.

---

HYPOTHETICAL theory too often oppoſes the progreſs of knowledge, by withdrawing the attention from facts, and diſcouraging a diſpoſition to obſerve the phænomena of diſeaſe, and the action of medicines, without which it is impoſſible to make any advances in medical ſcience. But, ſtrange as it may appear,

---

* In No. XVI. p. 504, this gentleman's name was, by miſtake, printed Bany.

appear, it is not among men, who devote a great portion of their time to reading and ſpeculation, that we find thoſe, who are moſt liable to grant this unwarrantable preponderance, to conjecture over facts. Men who profeſs to be governed entirely by experience, who affect to deſpiſe theory of every kind, however legitimate, and who indulge an unbounded ſcepticiſm about every new improvement, are the genuine ſlaves of hypotheſes, which have been long diſcarded from the creed of every enlightened practitioner. When ſuch men attempt to theorize, that is, to draw general inferences from what paſſes before their eyes, which they are forced to do as well as their better-informed brethren, their reaſonings are compoſed of a confuſion of hypothetical opinions and expreſſions, imbibed without examination in their early ſtudies, which, in the lapſe of years, while ſcience advances, preſerve a ſtrong and undivided power over their minds. They are the firſt to cenſure, and the laſt to adopt the ſucceſſive improvements in their profeſſion, and are the more dangerous to the cauſe of ſcience and humanity, becauſe they uſually poſſeſs a ſtock of arguments, which favour the natural indolence, and neceſſarily limited information, of the generality of mankind.

The opinions relative to the effect of heat and cold on the human body, which long prevailed in the ſchools, were intirely drawn from the analogy of unorganized matter, and the *vital principle* was overlooked, or wholly diſregarded. It gives me pain to obſerve, that the doctrine of relaxation, which is a neceſſary conſequence of this falſe analogy, is ſtill admitted by many practitioners, and has prevented the more general uſe of the warm bath, which, from the united teſtimony of phyſicians of the greateſt reſpectability, is not only a ſafe, but a moſt efficacious remedy. The following caſe, beſides poſſeſſing ſome claims to notice in other reſpects, muſt furniſh the advocates for this exploded hypotheſis, with a ſatiſfactory refutation, and may facilitate the application of it, in diſeaſes of extreme debility, where other means have been tried without ſucceſs. I hope this will be a ſufficient conſideration to excuſe its publication, as well as the previous remarks.

Miſs Ellen M'Sweeny,* æt. 21, July, 1799, after much faſting

* This lady belongs to a religious ſect, which in this part of Ireland, expects from its votaries, a rigid compliance with all the rules of the church. The ladies, as may naturally be ſuppoſed, are much more ſtrict in the obſervance of faſt days than the men, who differ very little in this reſpect from the Continental Catholics. It will ſcarcely be believed, but is notwithſtanding faſting and abſtinence during laſt Lent, ſat up for ſome nights with a friend, who was ill of a fever, which terminated fatally. Grief, fatigue, want of reſt, together with the previous debilitating effects of faſting, or a diet not ſufficiently nutritive, brought on ſlight hyſterics, which were followed by frequent vomiting after food or drink. I firſt ſaw her in the month of April laſt, which was about five or ſix weeks after the firſt attack. She complained of a dull pain in the region of the ſtomach, extending to the back; ſhe threw up every thing ſhe ſwallowed during the day, but ſometimes retained about a pint of whey at night, if ſhe dropped aſleep immediately after taking it. Her ſtrength and appearance were not yet materially altered; her pulſe was ſixty, countenance pale, belly coſtive, catamenia regular. I directed ſucceſſively, laudanum, æther, bitters, the ſaline julep, and Schweppe's Seltzer water; I alſo ordered a warm plaſter to be applied to the ſtomach, and ſome aloetic pills to obviate coſtiveneſs. I adviſed that ſhe ſhould take her food in ſmall quantities, and uſe gentle exerciſe on horſeback, which ſhe had previouſly tried with ſome apparent benefit. Her retaining the whey at night, ſuggeſted the idea of ſubſtituting broth, which anſwered for ſome nights, but was afterwards rejected as ſoon as taken.

Her friends perceiving her evidently growing worſe under this treatment, brought her to Cork, in May laſt, that ſhe might be under my immediate care, and have the advantage of additional advice. She was now much emaciated, her pulſe was weaker, and her general ſtrength very much diminiſhed; the pain of her back and ſtomach was increaſed; her legs ſwelled towards evening; ſhe ſtill rejected her food and drink without any apparent effort; ſhe had not above two hours ſleep at night, from which ſhe awoke unrefreſhed. At this time my friend, Dr. Sagrue, ſaw her with me, and ſuſpecting an affection of the liver, ſuggeſted the trial of mercury, which was uſed ſo as to excite ſlight ptyaliſm without any advantage. A bliſter to the ſtomach, and another to the back ſoon after, intirely removed the pain. Some internal remedies were given of the ſame nature with thoſe already mentioned; but perceiv-ing ing an undoubted fact, that many of the delicate young ladies of the town of Macromp, where Miſs M'Sweeny reſides, actually live on one meal a day, during the entire of Lent annually; and as meat is prohibited, and they ſeldom have an opportunity of getting fiſh, this meal is in general nothing more than bread and butter and tea. The conſequences may be eaſily imagined. It is to be hoped that a more liberal policy, with regard to the clergy of this extenſive ſect, will ſhortly put an end to this profligate waſte of health and life.

ing no benefit from them, ſhe was confined to port wine highly ſpiced, and broth, of which, by the moſt cautious management, ſhe was at length able to retain about four or five tea ſpoonsful of each in a day. But, whenever we attempted to exceed this quantity, the intire of what ſhe had taken for the day was inſtantly thrown up. She ſoon after returned to the country, without leaving her friends or her phyſicians the ſmalleſt hope of her recovery.

Having occaſion, in a ſhort time after, to viſit a patient in her neighbourhood, I called on her, and was really ſhocked at the ſudden alteration in her appearance. She was in every reſpect worſe, the weakneſs and emaciation were increaſed, her features were collapſed, and her ſkin was of a deadly paleneſs; the period had paſſed without the menſes appearing; ſhe had ſcarcely any ſleep; ſhe conſtantly vomited at night the whole of the broth and wine ſhe took in the courſe of the day. I had juſt read the extracts from Dr. Marcard's work, (Ueber den Gebrauch der baden) in Dr. Beddoes's valuable Eſſay on Conſumption, which though they did not exhibit any inſtance exactly ſimilar to that of my patient, yet furniſhed ſome analogical facts, not unfavourable to the trial of the warm bath in her caſe. From the manner in which the diſeaſe originated, and the extreme debility which it had induced, I derived ſome faint hopes from the gently ſtimulating effects of this remedy. The firſt trial, however, was rather diſcouraging. The bath was heated to about 98 degrees of Fahrenheit. She was ſeized with a vomiting the moment ſhe entered it, which ceaſed on her being taken out. She thought ſhe felt weaker the remainder of the day, and had frequent returns of coldneſs and ſhivering. Next day, the bath was made ſome degrees warmer, and ſhe continued in it a quarter of an hour, without the leaſt inconvenience. She felt warm and comfortable the intire day after. She retained a quantity of milk and tea on her ſtomach, and ſlept ſix hours that night. Encouraged by this trial, ſhe perſevered in the daily uſe of the bath, gradually increaſing the temperature of it, with the ſame beneficial effects. She ſoon gained ſufficient ſtrength to bear the exerciſe of the ſwing, which ſhe had before attempted, without ſucceſs. Her diet was milk, broth, jelly, tea, and coffee; and by my advice ſhe ſoon after added, bread, potatoes, cauliflowers, and artichokes, without any return of the vomiting. But ſhe found, on repeated trials, that meat in a ſolid form, however prepared, or ſeaſoned, would not remain a moment on her ſtomach. In about ſix weeks ſhe was ſo much recovered, as to think the bath no longer neceſſary, and by my advice ſhe diſcontinued it. I ſuſpect that rejection of meat in a ſolid form, depends

 rather rather on fome naufeating affociation, than on any remaining debility of the ftomach, as fhe retains and digefts fubftances, which are in general confidered as much more difficult of digeftion. She has lately made no trials with it, and yet her health is perfectly reftored, and fhe is even fatter and more blooming than ever.

I lament that the degree of the thermometer to which the bath was heated, was not daily afcertained. It muft have been greater than the ordinary temperature of warm baths, as her fifter affured me, that it was in general fo hot, that *fhe* could not bear her hands in it.

The above ftatement was drawn up at different periods, previous to October or November laft. I faw Mifs M'Sweeny a few days ago; fhe continues well, and very much difpofed to fat; fhe began to eat meat in laft month, and has continued it fince without any inconvenience.

There is another doctrine, which appears to be better founded, that has been objected againft the warm bath, viz. the danger of catching cold. A medical friend of mine, who unites an ardent love of improvement with the experience of a long life, has been many years in the habit of recommending the warm bath in cafes of great exhauftion from lues venerea, as well as to obviate the debilitating effects, occafioned by the immoderate exhibition of mercury, with the happieft fuccefs. He has affured me, that in fuch cafes he has never found it neceffary to injoin any precautions for avoiding cold. On the contrary, when the patient's ftrengh was fufficient, he always advifed him to go into the air after bathing, and never obferved any bad effects from it, even in cold weather. The luminous theory of Brown, fo happily explained and illuftrated by Dr. Beddoes, deferves attention on this fubject.

